# TMT Quantitative Proteomics Analysis Reveals the Effects of Transport Stress on Iron Metabolism in the Liver of Chicken

**DOI:** 10.3390/ani12010052

**Published:** 2021-12-28

**Authors:** Jun Liu, Dunhua Liu, Xun Wu, Cuili Pan, Shuzhe Wang, Lu Ma

**Affiliations:** 1 School of Agriculture, Ningxia University, Yinchuan 750021, China; dove_lj@126.com; 2 School of Food & Wine, Ningxia University, Yinchuan 750021, China; wuxun1232021@163.com; 3 Ningxia Key Laboratory of Ruminant Molecular and Cellular Breeding, School of Agriculture, Ningxia University, Yinchuan 750021, China; 13466552406@163.com (C.P.); wangshuzhe19921111@163.com (S.W.); 4 Department of Business Management, Shizuishan Institute of Industry and Trade, Shizuishan 753000, China; ma15109685640@163.com

**Keywords:** transport stress, broilers, iron homeostasis, TMT proteomics

## Abstract

**Simple Summary:**

Transport stress (TS) can impact the physiology and psychology of broilers, and this can be an important factor affecting liver iron metabolism in broilers. By establishing a transport model group, broilers (n = 144) reared under the same conditions were allocated into six groups and transported duration for 0, 0.5, 1, 2, 4, and 6 h. The results showed that the enrichment of iron content in the liver was the highest at a transport duration of 4 h, so the effect of transport duration of 4 h on iron metabolism was further investigated using TMT quantitative proteomic analysis. It was found that TS caused the enrichment of iron ions in the liver, TMT identified FTH1, IREB2, and HEPH as key proteins affecting iron metabolism, and key genes regulating iron homeostasis were validated using RT-PCR.

**Abstract:**

Abnormal iron metabolism can cause oxidative stress in broilers, and transport stress (TS) may potentially influence iron metabolism. However, the mechanisms by which TS affects iron metabolism are unclear. This study used quantitative proteome analysis based on tandem mass tag (TMT) to investigate the effects of TS on liver iron metabolism in broilers. Broilers (n = 24) reared under the same conditions were selected randomly into the transported group for 4 h (T2) and non-transported group (T1). Results showed that the serum iron level and total iron-binding capacity of broilers in the T2 were significantly higher than those in the T1 (*p* < 0.05). The liver iron content of broilers in the T2 (0.498 ± 0.058 mg·gprot^−1^) was significantly higher than that in the T1 (0.357 ± 0.035 mg·gprot^−1^), and the iron-stained sections showed that TS caused the enrichment of iron in the liver. We identified 1139 differentially expressed proteins (DEPs). Twelve DEPs associated with iron metabolism were identified, of which eight were up-regulated, and four were down-regulated in T2 compared with T1. Prediction of the protein interaction network for DEPs showed that FTH1, IREB2, and HEPH play vital roles in this network. The results provide new insights into the effects of TS on broilers’ liver iron metabolism.

## 1. Introduction

The pre-slaughter transport process is an important integral part of poultry management [1]. Pre-slaughtering transport of market-age broilers from their geographically dispersed farms is an unavoidable common practice [2]. During transit, chickens are exposed to numerous potential stressors, including handling, feed withdrawal, noise, vibration, thermal extremes, social disruption, crowding, and restriction of movement [3], which may lead to undesirable changes in animal welfare and immunity. The transport stress (TS) responses comprise mainly autonomic responses via activation of the autonomic nervous system (ANS) mediated by adrenaline and noradrenaline including increased respiration and heart rate, elevated body temperature, and promotion of energy utilisation from body reserves [4], accelerating glycogenolysis and suppressing energy storage [5]. In addition, birds are more sensitive to temperature than other monogastric animals due to feather coverage and the absence of sweat glands [6]. TS also increases the concentration of circulating corticosterone hormone, via activation of the hypothalamic–pituitary–adrenal (HPA) axis, which has a significant impact on the hepatic glycogen, protein and lipid metabolism, and meat quality [7].

The metabolic consequences of TS are mainly reflected in metabolic acidosis and oxidative stress, both of which lead to cytotoxicity, a free radical-mediated chain reaction [8]. The main source of ROS in tissues is the leakage of electrons in the mitochondrial respiratory chain during the conversion of molecular oxygen to water [9]. In addition, iron ions can promote the production of hydroxyl radicals. Evidence suggests that ROS were closely associated with iron because it could diffuse freely over cellular membranes and then interact with ferrous iron (known as Fenton reaction), which catalyzes a Fenton-type reaction to produce more reactive oxygen radicals [10]. Therefore, the condition of iron during pre-slaughter transport might be an important factor in the health of broilers and the quality of chicken meat. 

Iron, one of the essential trace elements for the growth and development of poultry, is utilized by most living cells and organisms for essential biochemical functions, such as oxygen transfer, DNA, RNA and protein synthesis, electron transfer, cellular respiration, cell proliferation and differentiation [11,12]. Iron is also involved in the function of catalases and peroxidases that protect the cells against the formation of free radicals, but iron concentration must be finely regulated because any excess of free iron is rapidly toxic. After all, iron is a transition metal with divalent and trivalent oxidation numbers and exerts its toxicity by catalyzing ROS generation. The most toxic hydroxyl radical (•OH) is produced in large quantities in the Fenton reaction. •OH may cause cell injury by inducing damage to the lysosomal, cytoplasmic, nuclear, and mitochondrial membranes, apoptosis through activation of the caspase cascade, and hyperoxidation of fatty acid chains [13]. Modern medical research has found that an imbalance in iron homeostasis can induce metabolic disorders and several diseases in the body, but this process is not limited to physiological processes [14]. Psychological stress can even activate the thalamic–pituitary–adrenal (HPA) axis system, causing an increase in serum adrenocorticotropic and adrenocorticotropic hormone levels and a decrease of serum iron levels, hepatic iron enrichment, and the development of iron overload [15,16].

At the individual level, the liver maintains iron homeostasis by balancing iron supply with iron utilization and losses. The liver orchestrates systemic iron balance by producing and secreting hepcidin, inducing degradation of the iron exporter ferroportin, which regulates the import of iron from the bloodstream to the liver [17]. The liver has been shown to be a hub for iron regulation, but the effect TS on hepatic iron metabolism is unknown. So, does TS affect changes in chicken liver iron content? If so, how is this iron being transported? What proteins are involved after the iron homeostasis is disrupted? The process involves a range of proteins, biological processes, and pathways. Due to the complexity of iron metabolism, information on proteins is needed to fully understand the biological processes and pathways. The quantitative proteomics approaches based on full tandem mass tag (TMT) have been used to study animal metabolic processes [18,19]. The analysis of this resource has enabled us to examine the mechanisms of protein diversification, thereby expanding knowledge of the complexity of TS-induced iron metabolism.

## 2. Materials and Methods

### 2.1. Experimental Design, Chickens, Management, and Transport Conditions

This study was approved by the Animal Care and Use Committee, Ningxia University. One-day-old male Chinese white-feathered broilers were obtained from a commercial hatchery and were reared until 72 d of age under normal conditions with free access to water and feed in the poultry farm of the Ningxia Haoshuichuan Breeding Co., Ltd. (Guyuan, China). 

Transport model group allocation: Seventy-two day-old Chinese, white-feathered broilers with consistent body weights (2.50 ± 0.10 kg) were randomly allocated to six treatment groups (n = 24 per group) with transport duration set at 0, 0.5, 1, 2, 4, and 6 h, respectively. Every 4 broilers were transported in a single crate with the dimensions of 52 × 43 × 26 cm (length × width × height). Fasting for 10 h, with access to water prior to transport, no drinking water was provided during transport. The truck was an opened truck designed for shipment of broilers, and the 36 crates were put randomly on the truck (4.8 m × 1.46 m × 0.95 m), with 3 rows × 3 columns × 4 layers and no apparent gaps among rows and layers. The pre-slaughter handling included catching the chickens, weighing, loading into containers, transport, unloading, weighing after the transport and slaughter. The 0 h broilers were slaughtered directly, and the rest were slaughtered after completing the transport time separately. Transport was undertaken on a ring road (approximately 78.81 km·lap^−1^) near the city of Yinchuan, and the average speed was 60 km·h^−1^. The truck’s average relative humidity and temperature were 34 to 45% and 30.1 to 35.6 °C, respectively. 

Distribution of broilers in T1 and T2 groups: Seventy-two-day-old Chinese, white-feathered broilers with consistent weights (2.50 ± 0.10 kg) were randomly allocated to two treatment groups (T1 and T2, n = 24 per group). T1 and T2 groups were set up slightly differently from the transport model group. The non-transport group (T1) were captured, loaded, and left in the transport vehicle for 4 h, and broilers in the transport group (T2) were captured, loaded, and transported for 4 h. At the end of transport, groups T1 and T2 broilers were slaughtered together. The pre-slaughter handling of the broilers, the transport vehicle, the speed of the vehicle, and the environment outside the vehicle were kept consistent for T1 and T2 with the transport model group.

### 2.2. Slaughter and Sample Collection

After transportation, blood samples were immediately collected from the jugular vein and put into 5 mL EDTA-evacuated tubes, which were then centrifuged at 2500× *g* for 15 min at 4 °C, frozen in liquid nitrogen, and plasma samples were stored at −80 °C until analysis. Broilers were slaughtered using electrical stunning and exsanguination from the jugular vein and de-feathered. After dissection, the livers were rapidly cleaned with PBS, and fresh samples were placed in 4% paraformaldehyde fixative for haematoxylin-eosin (HE) and Prussian blue (PB) analysis, placed in liquid nitrogen, and stored at −80 °C for proteomic and biochemical analysis [20].

### 2.3. Biochemical Indices of Plasma and Liver

Serum concentrations of lactate dehydrogenase, glucose, serum iron, and liver iron were measured using an Chemray 800 automated biochemistry analyzer (Rayto Life and Analytical Sciences Co., Ltd., Shenzhen, China) using colorimetric methods and following the instructions of the manufacturer of the corresponding reagent kit (Nanjing Jiancheng Bioengineering Institute, Nanjing, China). The concentrations of cortisol, corticosterone, glutathione peroxidase, and adrenocorticotropic hormone were determined with a commercial ELISA kit (Nanjing Jiancheng Bioengineering Institute, Nanjing, China) according to the manufacturers’ instructions.

### 2.4. Determination of Iron Metabolism-Related Indicators in Blood

A fully automated biochemical analyser (Myriad Biomedical Electronics Co., Shenzhen, China) was used to determine the serum’s transferrin-bound iron and unsaturated iron binding capacity. The total iron-binding capacity and transferrin saturation were then calculated, total iron binding capacity was expressed as the sum of serum iron content and unsaturated iron binding capacity, and transferrin saturation was expressed as serum iron content divided by total iron binding capacity, multiplied by 10,000.

### 2.5. Haematoxylin-Eosin (HE) and Prussian Blue (PB) Staining

The liver samples were fixed with 4% paraformaldehyde at room temperature. Fixed tissues were dehydrated in 30% sucrose (*v*/*v*), paraffin-embedded, and sectioned (10 μm) with a sliding microtome (Leica Mikrosysteme Vertrieb GmbH, Solms, Germany). Sections were stained with hematoxylin or Prussian blue solution, dehydrated with ethanol and xylene, sealed with neutral gum, and then examined under a light microscope (Olympus Corporation, New York, NY, USA) [21,22].

### 2.6. ROS Fluorescent Staining

Chicken livers were frozen, sectioned, and dried at room temperature (20 ± 1.5 °C). The sections were washed three times with PBS buffer (pH 7.4, 0.01 M) for 5 min each, followed by DAPI staining for 10 min in the dark to stain the nuclei, and the sections were then sealed with an anti-fluorescence quencher. The slices were observed under a fluorescence microscope, and images were collected (DAPI UV excitation at 330–380 nm, emission at 420 nm, blue light; FITC excitation at 465–495 nm, emission at 515–555 nm, green light; CY3 excitation at 510–560 nm, emission at 590 nm, red light) (Front-mounted Fluorescence Microscope Eclipse C1, Nikon Co., Tokyo, Japan).

### 2.7. TMT Proteomics Analysis

#### 2.7.1. Sample Preparation

The chicken liver tissues were ground in liquid nitrogen. Hence, 400 µL of SDT lysate buffer (4% SDS, 100 mM DTT, 150 mM Tris-HCl pH 8.0) was added to each sample, ultrasonicated for 2 min in an ice bath, and centrifuged at 16,000× *g* for 20 min at 4 °C. The supernatant was collected and quantified with a BCA Protein Assay Kit (Nanjing Jiancheng Bioengineering Institute, Nanjing, China).

#### 2.7.2. Protein Digestion

Digestion of protein (300 μg for each sample) was performed according to the FASP procedure [23,24]. Briefly, 200 µL of UA buffer (8 M Urea, 150 mM Tris-HCl, pH 8.0) were added to the protein sample, mixed well, followed by repeat ultrafiltration (Microcon units 30 kD, 12,000× *g* for 15 min) facilitated by centrifugation. Then, 100 μL 0.05 M iodoacetamide in UA buffer was added to block reduced cysteine residues, and the samples were incubated for 20 min in the dark. The filter was washed with 100 μL UA buffer three times and then 100 μL 25 mM NH_4_HCO_3_ twice. Finally, the protein suspension was digested with 4 μg trypsin (Promega, Madison, WI, USA) in 40 μL 25 mM NH_4_HCO_3_ overnight at 37 °C, and the resulting peptides were collected as a filtrate. The peptides were desalted using a desalting spin column (Thermo Fisher Scientific, Waltham, MA, USA) for quantification.

#### 2.7.3. TMT Labeling of Peptides

Briefly, 100 μg of the peptide was taken from each sample and labeled according to TMT labeling kit instructions (Thermo Fisher Scientific, Waltham, MA, USA). Each aliquot (100 μg of peptide equivalent) was reacted with one tube of TMT reagent. After the sample was dissolved in 100 μL of 0.05 M TEAB solution, pH 8.5, the TMT reagent was dissolved in 41 μL of anhydrous acetonitrile. The mixture was incubated at room temperature for 1 h. Then, 8 μL of 5% hydroxylamine were added to the sample and incubated for 15 min to quench the reaction. The multiplex labeled samples were pooled together and lyophilized. The peptides of each fraction were dried and solubilized with 0.1% fomic acid for LC-MS analysis.

#### 2.7.4. LC-MS Analysis

LC- MS analysis was performed on a Q Exactive mass spectrometer coupled to Easy nLC 1200 (Thermo Fisher Scientific, Waltham, MA, USA). Peptide from each fraction was loaded onto the C18-reversed-phase column (12 cm long, 75 μm ID, 3 μm) in buffer A (0.1% formic acid) and separated with a linear gradient of buffer B (95% acetonitrile) at a flow rate of 300 nL/min over 90 min. The linear gradient was set as follows: 0–2 min, linear gradient from 2% to 8% buffer B; 2–42 min, linear gradient from 8% to 28% buffer B; 42–47 min, linear gradient from 28% to 40% buffer B; 47–52 min, linear gradient from 40% to 100% buffer B; 52–60 min, buffer B maintained at 100%. The peptides were separated and analyzed by DDA (Data Dependent Acquisition) mass spectrometry using a Q-Exactive HF-X mass spectrometer (Thermo Fisher Scientific, Waltham, MA, USA). The analysis time was 60 min, detection mode: positive ion, parent ion scan range: 350–1800 m/z, primary mass resolution: 60,000 @m/z 200, AGC target: 3e6, primary Maximum IT: 50 ms. The peptide secondary mass spectra were acquired according to the following method: each full scan triggered the acquisition of secondary mass spectra (MS2 scan) of 20 highest intensity parent ions, Secondary mass resolution: 15,000 @m/z 200, AGC target: 1e5, Secondary Maximum IT: 50 ms, MS2 Activation Type: HCD (Higher energy collisioninduced dissociation), Isolation window: 1.2m/z, and Normalized collision energy: 1.2 m/z. 

#### 2.7.5. Database Searching and Analysis

The resulting LC-MS/MS raw data were imported into Maxquant (version 1.6.0.16, Thermo Fisher Scientific, Waltham, MA, USA) for data interpretation and protein search against the database Uni-prot_Hordeum-vulgare_201747–20180125 (downloaded on 25 January 2018, including 201,747 protein sequences), which was sourced from the protein database at https://www.uniprot.org/uniprot/?query=Hordeum-vulgare&sort=score (accessed on 22 December 2021). An initial search was set at a precursor mass window of 10 ppm. The search followed an enzymatic cleavage rule of Trypsin/P and allowed maximal two missed cleavage sites and a mass tolerance of 20 ppm for fragment ions. The modification set was as follows: fixed modification: Carbamidomethyl (C), TMT10plex(K), TMT10plex(N-term); variable modification: Oxidation(M) and Acetyl (Protein N-term). The minimum 6 amino acids for peptide, ≥1 unique peptides were required per protein. For peptide and protein identification, false discovery rate (FDR) was set to 1%. TMT reporter ion intensity were used for quantification. The database used for the search was Uni-prot_Gallus_gallus_34995_202108.fasta from the URL https://www.uniprot.org/taxonomy/9031/ (accessed on 22 December 2021) Protein Data Bank.

#### 2.7.6. Bioinformatics Analysis

NCBI BLAST + client software (https://www.ncbi.nlm.nih.gov/, accessed on 22 December 2021) and UniProtKB/Swiss-Prot (https://www.expasy.org/resources/uniprotkb-swiss-prot, accessed on 22 December 2021) were used to search the sequences of proteins. Differentially expressed proteins were screened with the cutoff of a ratio fold-change of >1.20 or <0.83 and *p*-values < 0.05 was carried out with Perseus software (Max-Planck-Institute of Biochemistry—Computational, Systems, Biochemistry) and Excel (Microsoft office 2019, Redmond, DC, USA) statistical computing software. Gene ontology (GO) terms were annotated using the software Blast2GO (BioBam Bioinformatics S.L., Valencia, Spain). After annotations, the proteins were blasted against the online Kyoto Encyclopedia of Genes and Genomes (KEGG) database (http://geneontology.org/, accessed on 22 December 2021). Hierarchical clustering analysis was conducted via R 4.1.2 (The University of Auckland, Auckland, New Zealand). GO and KEGG enrichment analyses were carried out with the Fisher’s exact test, and FDR correction for multiple testing was also performed. Enriched GO and Kegg pathways were nominally statistically significant at the *p* < 0.05 level. Protein-protein interactions were analyzed by string (http://string-db.org/, accessed on 22 December 2021) against the Sus scrofa database and considering a medium confidence score of 0.7 for interactions. 

### 2.8. Analysis of Gene Expression by Real-Time Quantitative PCR (RT-PCR)

Real-time PCR amplification was performed using an CFX fluorescent quantitative PCR instrument (Bio-Rad Laboratories, Hercules, CA, USA) in a total volume of 20 μL. The primer sequences are shown in Table S1. Actin was used as a reference gene. The PCR reaction mixture contained 4 μL 5× reaction buffer, 0.5 μL oligo (dT)18 primer (100 μM), 0.5 μL random hexamer primer (100 μM), 1 μL servicebio RT enzyme mix, and 110 μL total RNA. PCR amplification was performed using the initial heating for denaturation at 95 °C for 10 min, followed by 40 cycles at 95 °C for 15 s and 60 °C for 30 s. Then, the melting curve was analyzed at the end of every program [25]. Each PCR reaction was performed in triplicate. The 2^−ΔΔCT^ method was used to calculate each gene’s relative expression level [26].

### 2.9. Data Processing and Statistical Analysis

Results are presented as the means ± standard deviations (SD). All graphs were completed using Origin 2021 (Origin Lab Corporation, Northampton, MA, USA). Data were analyzed with one-way ANOVA using SPSS 25 (SPSS Inc., Chicago, IL, USA). A *p* < 0.05 was considered to be a statistically significant difference between the means.

## 3. Results

### 3.1. Changes in Blood Biochemistry and Liver Iron Content of Broilers in the Transport Model Group

As shown in Table 1, as the duration of transport increased in broilers of the transport model group, the concentrations of cortisol, corticosterone, adrenocorticotropic hormone, lactate dehydrogenase, glutathione peroxidase, glucose, and serum iron were increased (*p* < 0.05), indicating that the duration of transport affected the degree of stress, energy metabolism, and oxidative status in the broilers. It is interesting to note that the transport duration of 4 h resulted in the highest enrichment of iron in the liver. Therefore, further analysis of the metabolic status of iron ions in broilers at 4 h of transport was conducted. 

### 3.2. Changes in Iron Metabolism-Related Indicators in Blood and Tissue Iron

As shown in Table 2, serum iron content and total iron-binding capacity in T2 were significantly higher than T1, unsaturated iron binding capacity in T2 was significantly lower than T1, and transferrin saturation in T2 tended to be higher than T1 (*p* < 0.05). The liver iron content in T2 was significantly higher than T1 (*p* < 0.05). 

### 3.3. HE and PB Staining

**Figure 1:**
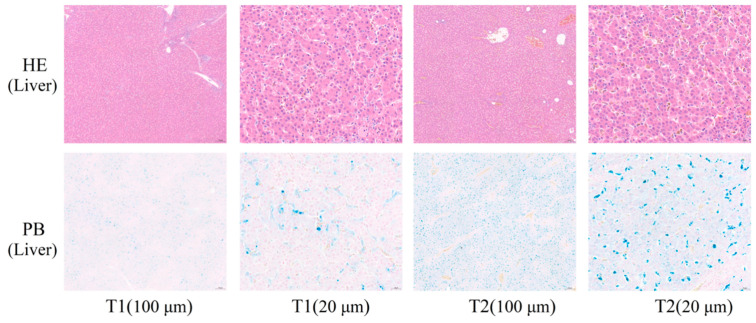
Morphological observations of broilers hepatic and chicken breasts. T1: not transported group (control); T2: transported group (for 4 h). HE, hematoxylin, and eosin staining; PB, Prussian blue staining; HE and PB staining, cells, and myogenic fibers are shown in red, and iron ions are shown in blue. The scale bar is 100 μm and 20 μm.

As shown in Figure 1, the liver cells in both T1 and T2 were clearly visible, well-arranged, and dense, with no pathological features, such as cell rupture and histolysis. Prussian blue staining caused the cells and tissues to appear pink and iron to appear blue, and it was noteworthy that the number and size of blue spots were higher in T2 than in T1 (Figure 1). This is consistent with the results shown in Table 2.

### 3.4. ROS Fluorescent Staining

**Figure 2:**
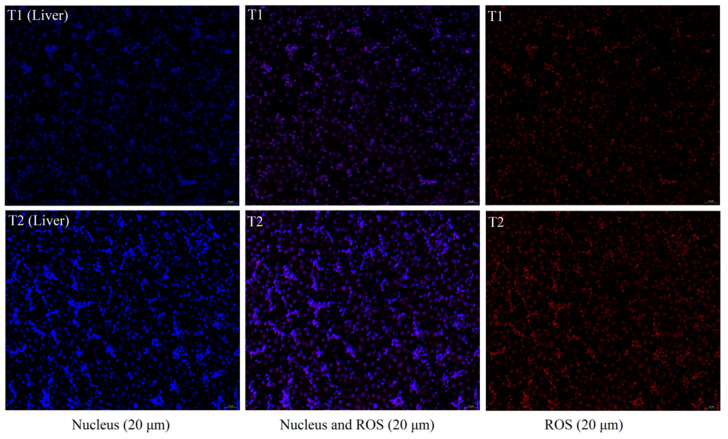
Fluorescent staining of liver and breasts for ROS. T1: not transported group (control); T2: transported group (for 4 h). Nuclei are shown in blue, and ROS are shown in red. The scale bar is 100 μm and 20 μm.

We assessed the effect of TS on ROS production in the liver (Figure 2). In contrast to the fluorescence imaging findings, where the ROS always accompanied the cell’s nucleus, the liver ROS fluorescence intensity in the T2 was significantly higher than in the T1.

### 3.5. Overview of the Chicken Liver Proteomic Analysis

**Figure 3:**
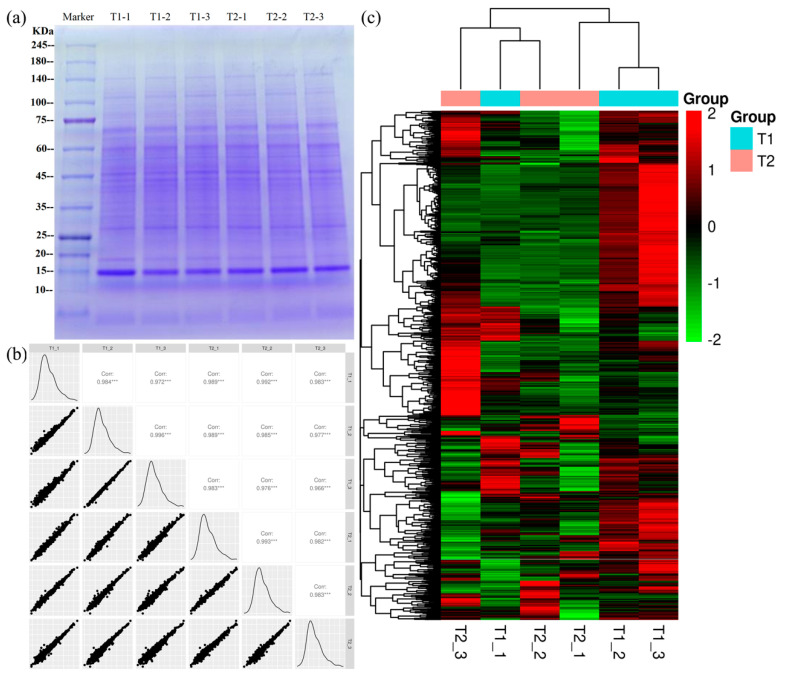
(**a**) SDS-PAGE gel electrophoresis protein profile of chicken liver. (**b**) Protein sample correlation plot (scatter plot). (**c**) Hierarchical clustering of differentially expressed proteins. The protein bands are distributed in a range of molecular weights of 10 to 245 kDa. “corr” is Pearson correlation analysis; “***” is a Pearson correlation greater than 0.8, which is a very strong correlation. The color scale bar located in the right, and green and red indicate decreased and increased levels of the identified proteins, respectively.

Chicken liver samples were used for protein extraction, followed by SDS-PAGE gel electrophoresis (1D gel). On the 1D pattern, the protein bands in different groups were similar (Figure 3a). Intra-group reproducibility was analyzed according to Pearson correlation, with R-values all greater than 0.8 (Figure 3b), indicating the reproducibility of the experiment. For comparison between T1 and T2 chicken liver samples, a protein exhibiting a fold change of >1.2 or <0.83 and a *p*-value of <0.05 was regarded as a differentially expressed protein (DEPs). Based on the two criteria, 1139 DEPs were identified. Moreover, the DEPs of each group were analyzed and displayed in the form of a hierarchical clustering heat map (Figure 3c, Table S2). 

### 3.6. Annotation and Functional Enrichment of DEPs

**Figure 4:**
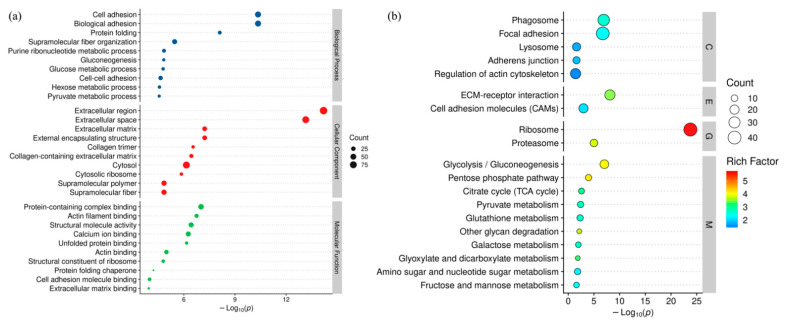
(**a**) GO function enrichment bubble chart. (**b**) KEGG pathway enrichment bubble map for DEPs.

To gain insights into the biological functions of DEPs, we performed GO functional enrichment analysis. The biological processes, cellular components, and molecular functions GO terms associated with DEPs are shown in Figure 4a, and the detailed data are shown in Table S3. These terms were associated with cell adhesion, biological adhesion, protein folding, supramolecular fiber organization, extracellular matrix, external encapsulating structure, protein-containing complex binding, actin filament binding, structural molecule activity, and calcium ion binding. KEGG enrichment analysis showed that DEPs were significantly enriched in the following functional categories: Phagosome, Focal adhesion, Ecm–receptor interaction, Cell adhesion molecules (CAMS), Ribosome, Proteasome, Glvcolvsis/Gluconeogenesis, and Pentose phosphate pathway (Figure 4b, Table S3).

### 3.7. Liver Iron Metabolism-Related Proteins and Protein-Protein Interactions (PPI) Network

**Figure 5:**
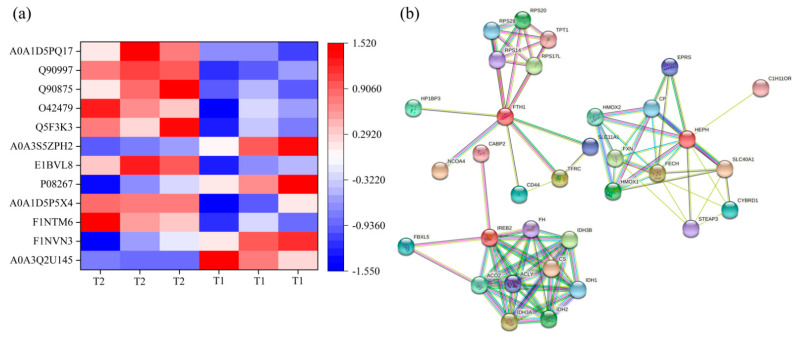
(**a**) Hierarchical clustering of DEPs associated with hepatic iron metabolism. (**b**) Protein-protein interaction network analysis. The color scale bar located in the right, and blue and red indicate decreased and increased levels of the identified proteins, respectively. FTH1: Ferritin, Heavy chain 1, TFRC: Transferrin receptor, SLC11A1: Natural resistance-associated macrophage protein 1, FECH: Ferrochelatase, HEPH: Hephaestin, SLC40A1: Solute carrier family 40 protein, IREB2: Iron responsive element binding protein 2, ACO2: Aconitate hydratase 1, CYBRD1: Cytochrome b reductase 1, STEAP3: Metalloreductase steap3, HMOX 1: Heme oxygenase 1, HMOX 2: Heme oxygenase 2.

To further analyze the effect of TS on hepatic iron metabolism, 12 DEPs associated with iron metabolism were identified, eight of which were up-regulated, and four down-regulated in T2 compared with T1 (Figure 5a, Table S4). Physical and functional protein-protein interactions (PPI) networks were constructed for the screened DEPs. Prediction of the protein interaction network for DEPs showed that FTH1, IREB2, and HEPH play vital roles in this network. Iron-responsive element-binding protein 2 (IREB2) had the highest number of interactions (Figure 5b).

### 3.8. Expression of Iron Homeostasis-Related Genes in the Liver of Broilers

**Figure 6:**
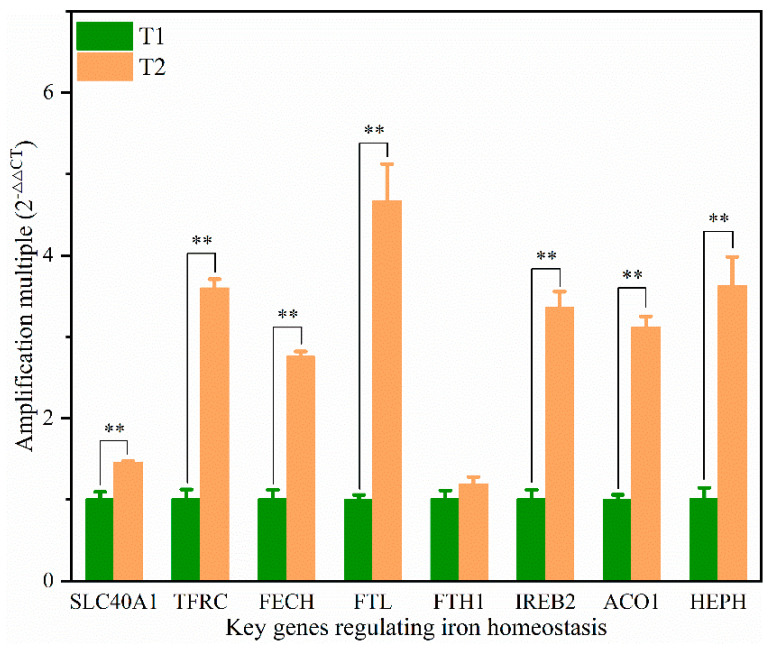
Expression levels of the key genes regulating iron homeostasis in the liver of broilers from T1 and T2. Actin was used as the internal reference. SLC40A1: Solute carrier family 40 protein, TFRC: Transferrin receptor, FECH: Ferrochelatase, FTL: Ferritin, Light polypeptide, FTH1: Ferritin, Heavy chain 1, ACO1: Aconitate hydratase 1, IREB2: Iron responsive element binding protein 2, HEPH: Hephaestin. ** is significant at *p* < 0.01.

We further investigated the expression of the genes associated with the regulation of iron homeostasis (Figure 6). Eight crucial genes related to iron homeostasis were analyzed. The mRNA expression levels of SLC40A1, TFRC, FECH, FTL, ACO1, IREB2, and HEPH in the T2 were significantly higher than those in the T1 (*p* < 0.05). The expression levels of FTL, TFRC, IREB2, and HEPH between T2 and T1 were up-regulated more than three-fold.

## 4. Discussion

Broilers can provide abundant, cheap, and nutritious animal proteins for human consumption [27]. The transport process of broilers before slaughter activates the autonomic nervous system (ANS), and the hypothalamic–pituitary–adrenal (HPA) responses of the body to promote corticosterone hormone secretion and accelerate anaerobic glycolysis. In this study, the levels of cortisol, corticosterone, and adrenocorticotropic hormone in the T2 were significantly higher than the T1 (Table 1). This stressful process could lead to an imbalance in iron homeostasis, increased ROS, and tumor necrosis factor (TNF) levels, and induce the apoptotic process [28,29]. There was evidence that these can interact with each other. For example, TNF increased labile iron level and subsequently promoted the production of mitochondrial ROS [8]. Serum iron levels were measured in T1 and T2 to determine if disorders in iron metabolism were present. Tissue iron content reflected the degree of iron enrichment in cells or tissues. Serum total iron-binding capacity and unsaturated iron binding capacity were indirect measures of transferrin concentration. The serum iron metabolism index showed a significant increase in iron content in the blood of broilers in T2 (*p* < 0.05) (Table 2), and the binding of transferrin was enhanced. The liver iron content in T2 was significantly higher than T1 (*p* < 0.05) (Table 2), which may be because hepatocytes are the primary iron storage cells in the body, and the liver is an essential organ in regulating iron homeostasis. Several studies have found that the transport process caused the release of iron ions, leading to an increase in serum iron level [30], which was consistent with the findings of this study.

**Figure 7:**
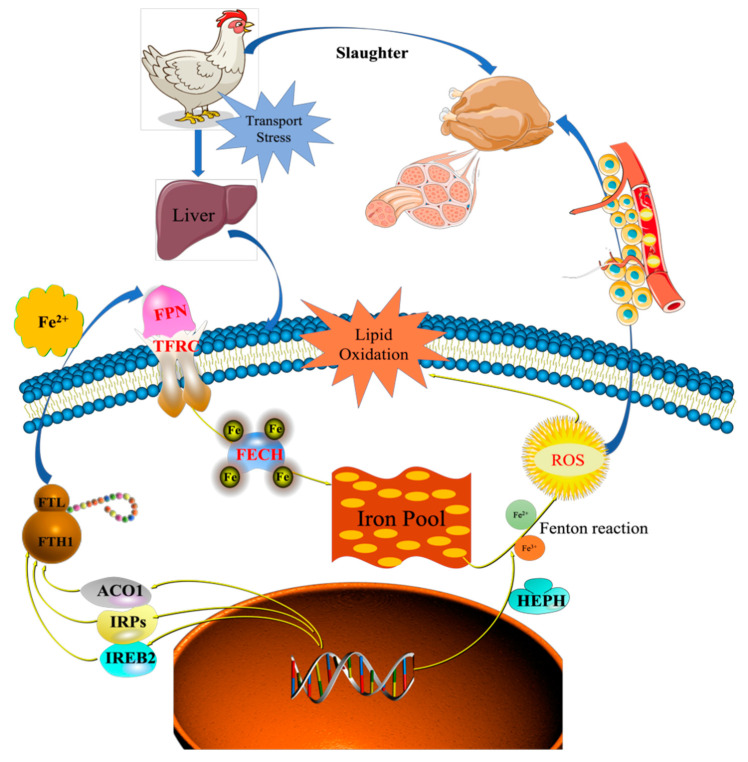
Proposed molecular mechanisms of transport stress-induced imbalance in iron homeostasis. SLC40A1, Solute carrier family 40 protein; TFRC, Transferrin receptor; FECH, Ferrochelatase; FTL, Ferritin, Light polypeptide; FTH1, Ferritin Heavy chain 1; ACO1, Aconitate hydratase 1; IREB2, Iron responsive element binding protein 2; HEPH, Hephaestin.

Iron homeostasis in the cells was regulated by balancing iron uptake with intracellular storage and utilization, and iron metabolism was visualized by mapping the pathways of iron ion transport (Figure 7). The process of iron uptake by the cell mainly involves the binding of transferrin receptors (TFRC) on the cell membrane to ferroportin (FPN), followed by endocytosis to transport iron into the cell or through divalent metal transporter 1 (DMT1) on the cell membrane to transport iron across the membrane into the cell [12,14]. In addition, the solute carrier family 40 protein (SLC40A1) regulates the ferrous iron transmembrane transporter activity and iron ion transmembrane transporter activity in the body. In the study, the expression of SLC40A1 was significantly higher in the T2 than T1 (Table S4, Figure 6) (*p* < 0.05). Hephaestin (HEPH), as a ferroxidase for Fe^2+^ to Fe^3+^ conversion, may be implicated in iron homeostasis and may mediate iron efflux associated with FPN. At the cellular level, FPN is regulated by the iron regulatory proteins (IRPs), iron-responsive element (IREB2), and cytoplasmic aconitate hydratase (ACO1). Once the iron entered the cell, it entered a hypothetical low-molecular-weight pool, otherwise known as the chelatable iron pool. At the same time, a large amount of ferrochelatase (FECH) was needed to chelate iron and keep the chelatable iron pool in a stable state [8,31]. In the study, the protein and mRNA expression levels of IREB2, TFR, and FECH in the T2 were significantly higher than those in T1 (Table S4, Figure 6), indicating that the ferric ion transport activity of FPN may be activated. The liver might regulate iron uptake or release, and there might be an imbalance in iron homeostasis. The elevated serum iron level led to the binding of ferroportin to FPN, which caused FPN to enter the cell, degrade it, and finally decrease the iron excretion. This is achieved predominantly at the level of protein synthesis (translation of mRNA into protein) rather than at the transcription level (mRNA synthesis). In the study, serum iron level in T2 was significantly higher than T1 (Table 2). The liver needs to absorb and transport iron ions to ensure stable serum iron levels. Iron transport requires the involvement of TRF, FPN, ferritin (light polypeptide), and ferritin heavy chain 1. Therefore, the expression of these genes was enhanced, which is consistent with our TMT quantitative proteomics and RT-PCR results.

Under normal physiological conditions, about one-third of transferrin is saturated with iron [31]. The buffering capacity of excess apo-transferrin ensures that each iron ion that enters the circulation remains shielded, and redox-active Fe^2+^ in the form of the labile iron pool (LIP) is maintained at low concentrations to sustain metabolic needs. In contrast, the excess is sequestered in proteins, including ferritin, to avert toxic repercussions [32]. However, when the organism is under physical or psychological stress, increased iron flux in the blood leads to gradual saturation of transferrin and accumulation of non-transferrin-bound iron. This is readily taken up by parenchymal tissue cells. The absorption process in the liver cells then causes an overload of iron in the liver. Under oxidative stress conditions, high levels of superoxide could induce Fe^2+^ release from iron compounds, including [4Fe-4S] cluster, heme, and ferritin, and cause iron-dependent accumulation of ROS. Interestingly, HEPH might function as a ferroxidase for Fe^2+^ to Fe^3+^ conversion [8,11]. The mRNA expression level of HEPH in the T2 was 3.61 times higher than in the T1, and the protein expression level was also up-regulated (Table S4, Figure 6). Iron could contribute to the ROS pool in the cell through the Fenton reaction in which Fe catalyzes the breakdown of H_2_O_2_ to yield hydroxyl radicals [19].

$${\mathrm{Fe}}^{2+}+H_{2}O_{2}\to {\mathrm{Fe}}^{3+}+{\mathrm{OH}}^{-}+\mathrm{OH}\cdot$$

Imbalance in the ROS generation and clearance rate may lead to oxidative stress and the consequent production of free radicals. Hydroxyl radicals are the most reactive free radical species and they may react with a wide range of cellular constituents including amino acid residues and attack membrane lipids to initiate a free radical chain reaction known as lipid peroxidation, leading to histopathological damage to hepatocytes [31]. In the study, liver iron content in the T2 was significantly higher than in the T1 (*p* < 0.05) (Table 2, Figure 2), which was consistent with the result of ROS (Figure 2). Histomorphological observations of the liver did not reveal significant histopathological damage to hepatocytes (Figure 1), which might be because the degree of imbalance in iron homeostasis was at a primary stage and caused little cellular damage. 

Transport could cause the excessive production and accumulation of ROS, ultimately result in oxidative stress [33]. We have verified by TMT proteomics and RT-PCR that iron may be a potential maker of ROS. In the present study, oxidative damage to cells during pre-slaughter transport of broilers could not be self-repaired because these chickens would be slaughtered without sufficient time to repair the oxidatively damaged cells. It has been suggested that ROS may affect meat quality by interfering with collagen turnover, and/or may cause the deterioration of meat by lipid peroxidation and protein oxidation directly through blood transport [34] and may even result in PSE meat [35]. Therefore, these DEPs in the current study might help reveal the genetic mechanism of transport stress-mediated imbalance of iron homeostasis using TMT quantitative proteomics strategies. Proteins are the executors of physiological functions and participate in the specific pathway to complete their biological functions comprehensively rather than independently performing their functions [36]. Moreover, some strategies are offered. If the composition of the basal diet of broilers is changed a week or more before slaughter, some natural phenolic-rich plant ingredients could be added to improve iron-induced oxidative stress by using the chelating properties of polyphenols with iron. The metabolic processes of iron in poultry farming should be taken into account, e.g., the excess of iron in the composition of the basal diet and the recovery process of newly hatched chicks after transfer, because these chicks are less able to adapt and recover from their environment.

## 5. Conclusions

In summary, the findings of this study suggest that iron metabolism was disturbed during pre-slaughter transport in broilers, especially in the liver. The imbalance in iron homeostasis induced the production of cellular ROS. We identified FTH1, IREB2, and HEPH as key proteins that regulate iron metabolism by TMT quantitative proteomics. This study contributes to understanding the complex biological processes controlling the imbalance of iron homeostasis mediated by TS and provides new insights into improving the adverse effects of TS in broilers.

## Figures and Tables

**Table 1:** Changes in blood biochemistry and liver iron content of broilers in the transport model group.

Variable	Transport Duration (h)					
	0	0.5	1	2	4	6
Cortisol (μg·L^−1^)	12.412 ± 0.105 ^a^	12.731 ± 0.223 ^b^	13.486 ± 0.469 ^c^	14.201 ± 0.249 ^d^	14.776 ± 0.324 ^e^	14.801 ± 0.209 ^f^
Corticosterone (μg·L^−1^)	76.563 ± 9.054 ^a^	117.034 ± 6.078 ^b^	128.129 ± 4.519 ^c^	140.402 ± 7.065 ^d^	145.605 ± 4.905 ^e^	123.407 ± 8.593 ^bcf^
Lactate dehydrogenase (U·L^−1^)	7.617 ± 0.378 ^a^	9.714 ± 0.478 ^b^	10.332 ± 0.104 ^c^	10.575 ± 0.127 ^d^	11.178 ± 0.342 ^e^	10.673± 0.258 ^def^
Glutathione peroxidase (U·L^−1^)	388.453 ± 18.349 ^a^	453.969 ± 25.448 ^b^	507.012 ± 18.349 ^c^	533.228 ± 10.408 ^d^	516.896 ± 5.908 ^e^	541.103 ± 31.348 ^cdef^
Adrenocorticotropic hormone (ng·L^−1^)	25.813 ± 1.273 ^a^	31.278 ± 3.279 ^b^	38.619 ± 1.003 ^c^	40.108 ± 1.887 ^cd^	39.774 ± 1.023 ^cde^	40.320 ± 0.953 ^cdef^
Glucose (nmol·L^−1^)	15.145 ± 0.473 ^a^	13.403 ± 0.113 ^b^	12.812 ± 0.207 ^c^	11.399 ± 0.194 ^d^	10.911 ± 0.098 ^e^	10.702 ± 0.134 ^f^
Serum iron content (μg·g^−1^)	0.110 ± 0.015 ^a^	0.135 ± 0.019 ^ab^	0.146 ± 0.012 ^bc^	0.167 ± 0.009 ^cd^	0.141 ± 0.015 ^bce^	0.152 ± 0.018 ^bcdef^
Liver iron content (mg·gprot^−1^)	0.359 ± 0.029 ^a^	0.314 ± 0.014 ^b^	0.306 ± 0.011 ^bc^	0.327 ± 0.104 ^abcd^	0.498 ± 0.058 ^e^	0.447 ± 0.089 ^aef^

^a–f^ Means with different letters within a row for the same indicator differed significantly between groups (*p <* 0.05).

**Table 2:** Effects of transport stress on serum iron metabolism-related parameters and tissue iron content in broilers.

Items	T1	T2
Serum iron metabolism index		
Serum iron content (μg·g^−1^)	0.110 ± 0.025 ^a^	0.140 ± 0.016 ^b^
Total iron binding capacity (μmol·L^−1^)	21.442 ± 3.531 ^a^	26.534 ± 2.058 ^b^
Unsaturated iron bonding capacity (μmol·L^−1^)	18.676 ± 3.883 ^a^	13.907 ± 2.103 ^b^
Transferrin saturation (%)	51.320 ± 5.884	53.480 ± 9.787
Tissue iron content		
Liver (mg·gprot^−1^)	0.357 ± 0.035 ^a^	0.498 ± 0.058 ^b^

^a,b^ Means with different letters within a row for the same indicator differed significantly between groups (*p* < 0.05). T1: not transported group (control). T2: transported group (for 4 h).

## Data Availability

Not applicable.

## Supplementary Materials

The following are available online at https://www.mdpi.com/article/10.3390/ani12010052/s1, Table S1: Primer sequences used for real time quantitative PCR, Table S2: TMT quantitative proteomics identifies all DEPs, Table S3: GO functional annotation and KEGG pathways of DEPs, Table S4: Identification of DEPs associated with iron metabolism.
